# Regulation of blood pressure and glucose metabolism induced by L-tryptophan in stroke-prone spontaneously hypertensive rats

**DOI:** 10.1186/1743-7075-8-45

**Published:** 2011-06-28

**Authors:** Ardiansyah, Hitoshi Shirakawa, Yuto Inagawa, Takuya Koseki, Michio Komai

**Affiliations:** 1 Laboratory of Nutrition, Department of Science of Food Function and Health, Graduate School of Agricultural Science, Tohoku University, 1-1 Tsutsumidori-Amamiyamachi, Aoba-ku, Sendai 981-8555, Japan; 2 Division of Food Management and Environmental Health, Department of Community Nutrition, Faculty of Human Ecology, Bogor Agricultural University, Jalan Kamper, FEMA Building W1-F3, Bogor 16680, Indonesia; 3 Laboratory of Food and Applied Life Sciences, Department of Food, Life, and Environmental Sciences, Faculty of Agriculture, Yamagata University, 1-23 Wakaba-machi, Tsuruoka 997-8555, Japan

## Abstract

**Background:**

Amino acids have been reported to act as modulators of various regulatory processes and to provide new therapeutic applications for either the prevention or treatment of metabolic disorders. The purpose of the present study is to investigate the effects of single oral dose administration and a continuous treatment of L-tryptophan (L-Trp) on the regulation of blood pressure and glucose metabolism in stroke-prone spontaneously hypertensive rats (SHRSP).

**Methods:**

First, male 9-week-old SHRSP were administered 100 mg L-Trp·kg^-1 ^body weight in saline to the L-Trp group and 0.9% saline to the control group via a gastric tube as a single oral dose of L-Trp. Second, three groups of SHRSP were fed an AIN-93M-based diet supplemented with L-tryptophan (L-Trp) (0, 200, or 1000 mg·kg^-1 ^diet) for 3 weeks as continuous treatment of L-Trp.

**Results:**

Single oral dose administration of L-Trp improved blood pressure, blood glucose, and insulin levels. Blood pressure, blood glucose, and insulin levels improved significantly in the L-Trp treatment groups. The administration of L-Trp also significantly increased plasma nitric oxide and serotonin levels.

**Conclusion:**

L-Trp by both single oral dose administration and continuous treatment improves glucose metabolism and blood pressure in SHRSP.

## Introduction

Our modern lifestyle is prone to overnutrition and lack of physical activity, which dramatically increases the incidence of metabolic disorders, including diseases associated with obesity, diabetes mellitus, dyslipidemia, and hypertension [1]. Currently, much effort is being invested in detecting bioactive compounds in foods that can decrease the risk of metabolic disorders, including dietary antioxidants such as vitamin E, vitamin C, carotenoids, and polyphenols, which are known to decrease the risk factors of cardiovascular diseases [2]. Taking a different approach to prevent metabolic disorders, nutritionists and food technologists have recommended several strategies, such as the intake of functional foods, food supplements, and medicines [3].

Recent research has demonstrated that certain amino acids, such as L-tryptophan (L-Trp), can regulate physiological processes and may be the basis for novel therapeutics to prevent or treat chronic diseases. Previous studies have shown that L-Trp lowers blood pressure (BP) and affects the level of serotonin in the brains of spontaneously hypertensive animals [4]; however, the relationship between decreased BP and serotonin is unclear. In addition, L-Trp affects glucose metabolism in rat hepatocytes [5].

Serotonin is a monoaminergic neurotransmitter that affects vasoconstriction, intestinal motility, primary hemostasis, liver repair, and the control of the T cell-mediated immune system. The first step in the synthesis of serotonin from tryptophan is the enzyme tryptophan hydroxylase, which is also the rate-limiting enzyme in serotonin's biosynthesis [6-9]. In animals, the effect of serotonin levels on glycemia is unclear. Studies have reported that an increase in serotonin concentrations can decrease blood glucose levels via the induction of insulin secretion in mice [10,11], but another study showed that serotonin may have a hyperglycemic effect [12]. The exact mechanism underlying this hypoglycemic effect is not well understood, although it may be mediated by the serotonin receptor in rats [13].

As a result, the purpose of the present study is to investigate the effects of single oral dose administration and a continuous treatment of L-tryptophan (L-Trp) on the regulation of blood pressure and glucose metabolism in stroke-prone spontaneously hypertensive rats (SHRSP), an animal model of hypertension-related disorders and insulin resistance in humans [14,15]. Specifically, we determined whether single oral dose administration or continuous treatment of L-Trp affected the blood pressure and glucose metabolism in these rats. This study further elucidated the underlying mechanisms of the effects of L-Trp on the parameters of metabolic disorders in SHRSP.

## Methods

### Animals, experimental design, and ethical guidelines

Male Izumo strain SHRSP was purchased from Japan SLC, Inc. (Shizuoka, Japan). The rats were housed in individual stainless steel cages in a controlled environment (23 ± 2°C, 50 ± 10% humidity, and 12-h light-dark cycle). All experimental procedures in this study were conducted in accordance with Japanese government guidelines (2005) and approved by the Animal Research-Animal Care Committee of Tohoku University (No. 20-dounou-21). This committee also supervised the care and use of the rats in this study.

### Blood pressure measurements

BP was measured with a BP meter (MK-2000, Muromachi Kikai, Tokyo, Japan) by using the tail cuff method without warming, as described in a previous study [16]. At least six measurements were taken for each rat. The individual systolic BP was calculated as the mean of four consistent readings of the systolic BP.

### Treatment with a single oral dose of l-tryptophan

After 1 week of acclimatization, 9-week-old rats were divided into a control group and an L-Trp group. After fasting, the rats for 16 h, L-Trp was dissolved in 0.9% saline and 100 mg L-Trp·kg^-1 ^body weight was orally administered via a gastric tube to the L-Trp group, while 1 ml of 0.9% saline was administered to the control group. BP was measured and blood samples from tail veins were collected before and 1, 2, 4, and 6 h after treatment for blood glucose and insulin analyses. After 1 week, the rats were treated again in the same manner, anesthetized with diethyl ether 4 or 6 h later, and then sacrificed. Finally, livers were dissected for mRNA analysis.

### Continuous treatment with l-tryptophan

We divided 9-week-old SHRSP into three groups and fed them an AIN-93M-based diet for 3 weeks. The diet of the control group was not supplemented, whereas the diets of the other two groups were supplemented with L-Trp at 200 mg·kg^-1 ^(LT200) or 1000 mg·kg^-1 ^(LT1000). The rats were allowed to consume these diets and drink water *ad libitum*. Their food intake was recorded every day. Each week, their systolic BP and body weight were measured, and blood samples from their tail veins were collected for blood glucose and insulin analyses. At the end of the experiment, the rats were anesthetized with diethyl ether after 16 h of fasting and then sacrificed. Immediately afterward, blood was collected from the abdominal aorta, and plasma was separated by centrifugation and stored at -20°C until analysis. For mRNA analysis, the liver of each rat cut into 0.5 cm sections then immediately transferred into a tube containing RNA-later solution and incubate at 4°C over night prior to storing the samples at -20°C as the manufacturer's instruction (Applied Biosystems Co., Tokyo, Japan).

### Analytical procedures

Plasma levels of total cholesterol, triacylglycerol, NEFA, HDL-cholesterol, glucose, blood urea nitrogen, and creatinine levels were measured by colorimetric enzymatic methods (Wako Pure Chemical Co.). The LDL-cholesterol level was calculated according to the Friedewald formula: LDL-C = (TC - HDL-C) - (1/5 × TG) [17]. Plasma insulin levels were measured using a rat insulin ELISA kit (Shibayagi Co., Gunma, Japan). Similarly, plasma serotonin levels were determined using an ELISA kit (Immunotech Co., Marseille, France). The nitric oxide (NO) level in the plasma was quantified using a colorimetric NO_2_/NO_3 _Assay Kit-C II (Dojindo, Kumamoto, Japan), which is based on the Griess method, as described in a previous study [18]. We also estimated insulin resistance (IR) with the homeostatic model assessment-insulin resistance (HOMA-IR) index, which is defined as HOMA-IR = (fasting insulin level (μU·ml^-1^) × fasting glucose level (mmol·l^-1^))/22.5 [19].

### RNA preparation and quantitative real-time polymerase chain reaction

Total RNA was isolated from liver samples using Isogen, a guanidine isothiocyanate-based reagent (Nippon Gene, Japan). The amount and purity of the isolated RNA were determined by spectrophotometric analysis at 260 and 280 nm (ratio of 260 nm/280 nm ≥ 1.9) and agarose gel electrophoresis (ratio of 28S RNA/18S RNA ≥ 1.5). Five micrograms of total RNA were used as a template to synthesize the cDNA. The RNA was denatured using oligo-dT, random primers, and 10 mM dNTPs (Amersham Biosciences, Tokyo, Japan) at 65°C for 5 min. Then, the RNA was added to a 20-μl reaction that contained 50 mM Tris-HCl buffer (pH 8.3), 0.1 M dithiothreitol, 50 units SuperScript III reverse transcriptase (Invitrogen, Carlsbad, CA, USA), and 20 units RNaseOUT RNase inhibitor (Invitrogen). This reaction was incubated at 25°C for 5 min, 50°C for 60 min, and 70°C for 15 min. Aliquots of the cDNA product were used as the template for quantitative real-time polymerase chain reaction (RT-PCR) analysis, which was performed using an Applied Biosystems 7300 Real Time PCR System (Foster City, CA, USA) and SYBR Premix Ex Taq (Takara Bio Inc., Shiga, Japan). The relative gene expression levels were normalized to the amount of eukaryotic elongation factor-1α1 (*EF-1*) mRNA [20]. The expression of EF-1 was unaffected by group treatment. The primer sequences were as follows: *EF-1*, 5'-GATGGCCCCAAATTCTTGAAG-3' (forward) and 5'-GGACCATGTCAACAATTGCAG-3' (reverse); glucokinase (*Gck*), 5'-GTGGCAATGGTGAACGACAC-3' (forward) and 5'-AATGTCGCAGTCGGTGACAG-3' (reverse); phosphoenolpyruvate carboxykinase 1 (*Pck1*), 5'-GATGACATTGCCTGGATGAAGTTT-3' (forward) and 5'-TGGGTTGATGGCCCTTAAGT-3' (reverse); fructose bisphosphatase 1 (*Fbp1*), 5'-CCACCAGAAGGCACCAGTTATC-3' (forward) and 5'-AACTCCTGCACGTCTTCGGT-3' (reverse); liver-type pyruvate kinase (*Pklr*), 5'-GGCAGATGATGTGGATCGAAG-3' (forward) and 5'-GCCAACCTGTCACCACAATC-3' (reverse). Amplified DNA was purified by polyacrylamide gel electrophoresis and cloned into pT7 blue cloning vector. After transmission in *E.coli*, cloned DNA fragment was sequenced by using ABI dye terminator sequencing kit and ABI310 DNA sequencer.

### Data analysis

Results were expressed as means ± SEM. Statistical analyses were performed with ANOVA and Fisher's tests. The P value cutoff for statistical significance was defined as P < 0.05. For the single dose experiment, the differences between group means were evaluated by Student's *t *test (SPSS statistical package, version 11.0). Differences were considered statistically significant when P < 0.05.

## Results

### Treatment with a single oral dose of l-tryptophan

**Figure 1:**
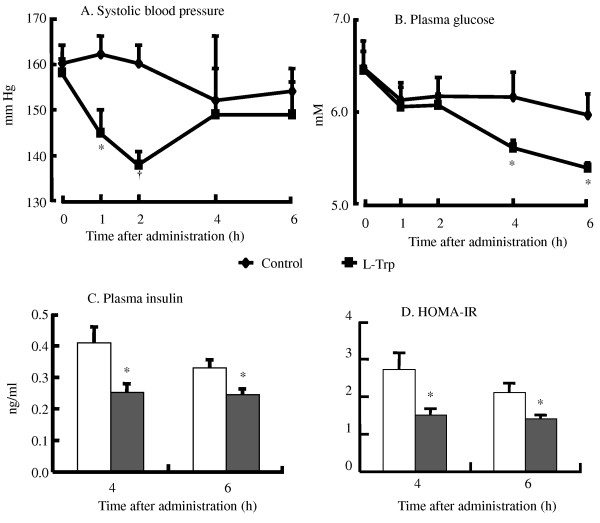
**A single oral dose effect of L-tryptophan (100 mg·kg^-1 ^body weight) on systolic blood pressure (A), blood glucose (B), plasma insulin (C), and homeostatic assessment-insulin resistance (HOMA-IR) index (D) 4 or 6 h after treatment in stroke-prone spontaneously hypertensive rats (SHRSP)**. N = 4 for all groups. Values are ME ± SEM. *P < 0.05, ^†^P < 0.01, compared with the control group.

We determined whether a single oral dose of L-Trp could lower high BP and high blood glucose levels in SHRSP. In the control group, after the administration of saline, the systolic BP in SHRSP remained almost constant for 6 h. In contrast, in the treatment group, these levels decreased 1 and 2 h after the administration of saline and L-Trp and then returned to basal levels in the control group after 4 h (Figure 1A). In addition, 4 and 6 h after treatment, blood glucose levels decreased significantly (Figure 1B). Consistent with this result, plasma insulin levels were also significantly lower at the same time points (Figure 1C). Moreover, 4 and 6 h after treatment, the HOMA-IR indices of the L-Trp group were significantly lower than those of the control group (Figure 1D). The effects of a single dose of L-Trp on the level of hepatic mRNA expression are given in Additional file 1, Table S1: A single oral dose effect of L-Trp on hepatic gene expression levels expressed as relative changes determined by quantitative RT-PCR. A single oral dose of L-Trp did not significantly alter the mRNA expression of all of the genes tested.

### Continuous treatment with l-tryptophan

**Figure 2:**
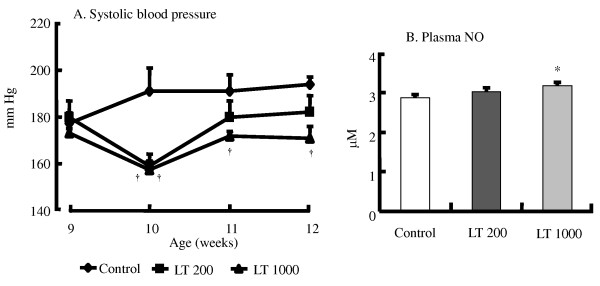
**Continuous treatment effect of L-tryptophan on systolic blood pressure (A) and plasma nitric oxide (NO) level (B) in SHRSP**. N = 5 for all groups. Values are ME ± SEM. *P < 0.05, ^†^P < 0.01, compared with the control group. C, control diet; LT200, diet supplemented with 200 mg·kg^-1 ^L-tryptophan; LT1000, diet supplemented with 1000 mg·kg^-1 ^L-tryptophan.

**Figure 3:**
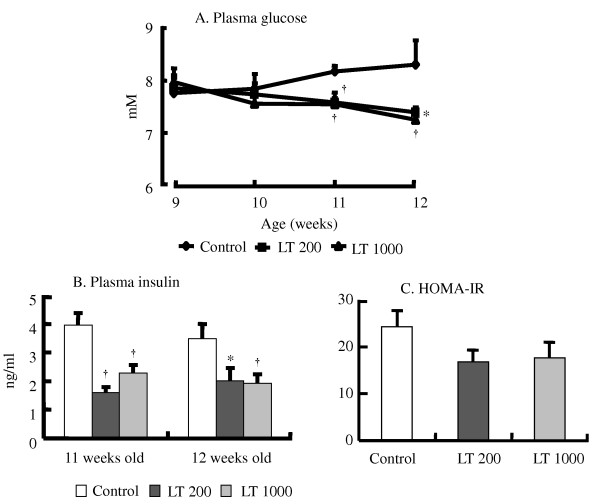
**Continuous treatment effect of L-tryptophan on plasma glucose (A) and insulin (B) levels in 11- and 12-week-old rats, and HOMA-IR indices (C) in 12-week-old rats**. N = 5 for all groups. Values are ME ± SEM. * P < 0.05, ^†^P < 0.01, compared with the control group. C, control diet; LT200, diet supplemented with 200 mg·kg^-1 ^L-tryptophan; LT1000, diet supplemented with 1000 mg·kg^-1 ^L-tryptophan.

**Figure 4:**
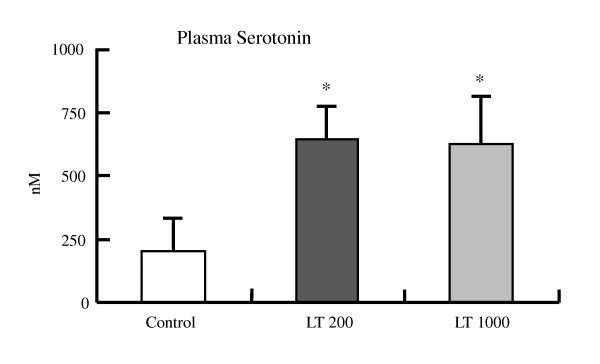
**Continuous treatment effect of L-tryptophan on plasma serotonin levels in SHRSP**. N = 5 for all groups. Values are ME ± SEM. ^†^P < 0.01 compared with the control group. C, control diet; LT200, diet supplemented with 200 mg·kg^-1 ^L-tryptophan; LT1000, diet supplemented with 1000 mg·kg^-1 ^L-tryptophan.

At the end of the experimental period, there were no differences in the final body weight, body weight gain, or daily food intake (Additional file 2, Table S2: Effect of diet on food intake, weight gain, and final body weight). The intake of L-Trp was 3.92 ± 0.08 and 19.67 ± 0.59 mg·d^-1 ^in the LT200 and LT1000 groups, respectively. Furthermore, the plasma and hepatic lipids and renal function parameters were similar among the three groups (data not shown). However, continuous treatment with L-Trp lowered BP. In the LT1000 group, the systolic BP of SHRSP was significantly lower than that of the control rats during the treatment period (Figure 2A); however, in the LT200 group, the systolic BP of the SHRSP significantly decreased after 1 week of treatment. Consistent with this result, the plasma NO level was significantly higher in the LT1000 group but not in the LT200 group (Figure 2B). We also analyzed blood glucose levels during the treatment period. In both treatment groups, L-Trp significantly improved blood glucose levels after 2 or 3 weeks of treatment (Figure 3A). Similar to the single dose of L-Trp, plasma insulin levels also significantly decreased after 2 or 3 weeks of treatment (Figure 3B). Also the HOMA-IR index in Figure 3C was not significantly modified by continuous treatment with L-Trp. In addition, plasma serotonin levels in the two L-Trp groups were higher than those in the control group (Figure 4). We also measured the hepatic mRNA expression levels of *Gck*, *Pck1*, *Fbp1*, and *Pklr *(Additional file 3, Table S3: Continuous treatment effect of L-tryptophan on hepatic gene expression levels expressed as relative changes determined by quantitative RT-PCR). The mRNA expression levels of Gck, *Pck1*, *Fbp1*, and *Pklr *in both L-Trp groups and the level of *Gck *in the LT1000 group did not significantly differ from those in the control group.

## Discussion

Our results demonstrated that both single oral dose administration and continuous treatment of L-Trp improves hypertension and hyperglycemia in SHRSP. This is the first study about the potential efficacy of oral L-Trp to prevent metabolic disorders using SHRSP as an animal model of severe hypertension and multisystem end-organ damage, especially in the kidney [21].

In the continuous treatment experiment, we found that the LT1000 group exhibited significantly lower BP one week after the administration of L-Trp. This effect was more pronounced in the LT1000 group than in the LT200 group, which only showed a weak antihypertensive effect; these data suggest that the effect of L-Trp may be dose-dependent. However, the blood glucose, insulin, and serotonin levels were almost the same in both L-Trp groups, so L-Trp did not have a dose-dependent effect on these levels. The underlying mechanism for the absence of a dose-dependent effect is not known; however, the lack of these effects may be due to the nature of *in vivo *experiments, which are more complex and therefore make it difficult to identify dose-dependent effects compared to *in vitro *experiments.

We observed that continuous treatment of 1000 mg·kg^-1 ^L-Trp significantly increased plasma NO levels (Figure 2B), which was consistent with the antihypertensive effect on SHRSP (Figure 2A). Most likely, enhanced plasma NO levels result in the vasodilator response of L-Trp. Our results are consistent with previous studies, which show that certain peptide substances significantly affect NO production and cause vasodilation [22,23]. In addition, we previously inferred that the increased plasma NO level is a consequence of the antihypertensive effect in SHRSP [18,23]. As a result, we concluded that L-Trp might enhance vasodilation in SHRSP; however, further investigation is needed to elucidate the underlying mechanism.

Since serotonin is synthesized from L-Trp, we tested whether plasma serotonin levels mediated the effect of L-Trp on blood glucose, insulin levels, and BP. Our results indicate that increased plasma serotonin levels in the L-Trp groups (Figure 4) might cause significantly reduced blood glucose levels and BP in SHRSP. Many previous studies have shown that serotonin induces a hypoglycemic effect by stimulating the 5-HT_7 _receptor in the rat adrenal gland, enhancing β-endorphin release and glucose uptake in skeletal muscle [11,24,25]. A recent study showed that a lack of serotonin in the β cells of *Tph1*^-/- ^transgenic mice reduces insulin secretion [26]. This finding suggests that serotonin is involved in the clinical progression of diabetes and may be the basis of novel anti-diabetic treatments. Our results are consistent with a previous study that demonstrated that the decrease in BP after the administration of L-Trp is mediated by serotonin [27]. Furthermore, our results imply that L-Trp can improve the regulation of glucose metabolism and blood pressure via plasma serotonin levels.

Our results have shown the evidence that L-Trp supplementation can increase insulin sensitivity followed by decreasing plasma insulin levels in single oral dose and continuous treatment of L-Trp (Figures 1C and 3B). As shown by the HOMA-IR indices, L-Trp supplementation increased insulin sensitivity more than the control diet (Figures 1D and 3C). Therefore, since SHRSP is animal model shows insulin resistance [15], the decreasing of plasma glucose and insulin levels in both studies and HOMA-IR of single oral dose of L-Trp administration indicates that L-Trp supplementation contributes to increase insulin sensitivity of the rats. To elucidate the mechanism of these physiological effects, we measured the effect of L-Trp on the hepatic mRNA expression levels of enzymes that are involved in glyconeogenesis and glycolysis, such as *Gck*, *Pck1*, and *Fbp1*. The mRNA levels of Gck were not significantly altered in this study. The expression of Gck was not even increased with LT1000 treatment.

In conclusion, we determined that single oral dose administration or continuous treatment of L-Trp reduced blood pressure and improved insulin sensitivity in SHRSP. Our results suggest that the effect of L-Trp on improving the physiological and biochemical parameters of metabolic disorders may be mediated by increased plasma serotonin levels. These results suggest that L-Trp might be a potential functional food supplement to help prevent metabolic disorders.

## List of abbreviations

BP: blood pressure; Fbp1: fructose bisphosphatase1; Gck: glucokinase; L-Trp: L-tryptophan; NO: nitric oxide; Pck1: phosphoenolpyruvate carboxykinase 1; Pklr: liver-type pyruvate kinase; SHRSP: stroke-prone spontaneously hypertensive rats.

## Conflicts of interest

The authors declare that they have no competing interests.

## Authors' contributions

AA performed most of the analytical work, designed the experiments, and wrote the final manuscript. YI contributed to the plasma serotonin measurements and some mRNA gene expression analyses. TK assisted with the experiments and participated in the discussion of the experimental results. HS and MK supervised the design of some of the experiments and drafted the manuscript. All authors are responsible for the content of the manuscript.

## Authors' information

Ardiansyah's second email address is ardy@biochem.tohoku.ac.jp.

## Supplementary Material

Additional file 1 **A single oral dose effect of L-tryptophan on hepatic gene expression levels expressed as relative changes determined by quantitative RT-PCR**. The effects of a single dose of L-Trp on the level of hepatic mRNA expression Click here for file

Additional file 2 **Effect of diet on food intake, weight gain, and final body weight**. Effect continuous treatment of L-Trp on final body weight, body weight gain, or daily food intake Click here for file

Additional file 3 **Continuous treatment effect of L-tryptophan on hepatic gene expression levels expressed as relative changes determined by quantitative RT-PCR**. Effect of continuous treatment of L-Trp on hepatic mRNA expression levels of *Gck*, *Pck1*, *Fbp1*, and *Pklr* Click here for file
