# Supplementary material for: Regulation of blood pressure and glucose metabolism induced by L-tryptophan in stroke-prone spontaneously hypertensive rats
Source: Nutr Metab (Lond). 2011 Jun 28;8:45. doi: 10.1186/1743-7075-8-45 (PMC3152873; doi:10.1186/1743-7075-8-45)
Supplement: Additional file 1 — A single oral dose effect of L-tryptophan on hepatic gene expression levels expressed as relative changes determined by quantitative RT-PCR. The effects of a single dose of L-Trp on the level of hepatic mRNA expression [file 1743-7075-8-45-S1.DOC]

Table S1. A single oral dose effect of l-tryptophan on hepatic gene expression levels expressed as relative changes determined by quantitative RT-PCR.^1^

| Gene | Control (n=4)  (ME ± SEM) | l-tryptophan (n=4)  (ME ± SEM) |
| --- | --- | --- |
| 4 h | | |
| *Gck* | 1.0 ± 0.1 | 2.0±0.7 |
| *Pck1* | 1.0 ± 0.1 | 1.2±0.1 |
| *Fbp1* | 1.0 ± 0.1 | 0.9±0.1 |
| *Pklr* | 1.0 ± 0.1 | 1.2±0.1 |
| 6 h | | |
| *Gck* | 1.0 ± 0.5 | 2.0±0.3 |
| *Pck1* | 1.0 ± 0.1 | 1.2±0.1 |
| *Fbp1* | 1.0 ± 0.1 | 1.3±0.2 |
| *Pklr* | 1.0 ± 0.1 | 1.1±0.1 |

^1^mRNA expression (fold); *Gck*, glucokinase; *Pck1*, phosphoenolpyruvate carboxykinase 1; *Fbp1*, fructose bisphosphatase 1; *Pklr*, liver-type pyruvate kinase.
