# Supplementary material for: Regulation of blood pressure and glucose metabolism induced by L-tryptophan in stroke-prone spontaneously hypertensive rats
Source: Nutr Metab (Lond). 2011 Jun 28;8:45. doi: 10.1186/1743-7075-8-45 (PMC3152873; doi:10.1186/1743-7075-8-45)
Supplement: Additional file 2 — Effect of diet on food intake, weight gain, and final body weight. Effect continuous treatment of L-Trp on final body weight, body weight gain, or daily food intake [file 1743-7075-8-45-S2.DOC]

Table S2. Effect of diet on food intake, weight gain, and final body weight

|  | Control (n=5)  (ME ± SEM) | LT200 (n=5)  (ME ± SEM) | LT1000 (n=5)  (ME ± SEM) |
| --- | --- | --- | --- |
| Food intake (g⋅d^-1^) | 18.8 ± 0.5 | 19.6 ± 8.5 | 19.7 ± 0.6 |
| Weight gain (g) | 50.8 ± 3.4 | 59.8 ± 2.6 | 58.3 ± 2.9 |
| Final body weight (g) | 274.6 ± 3.9 | 284 ± 5.9 | 283.2 ± 5.2 |

LT200, diet supplemented with 200 mg⋅kg^-1^ l-tryptophan; LT1000, diet supplemented with 1000 mg⋅kg^-1^ l-tryptophan.
