# Supplementary material for: Regulation of blood pressure and glucose metabolism induced by L-tryptophan in stroke-prone spontaneously hypertensive rats
Source: Nutr Metab (Lond). 2011 Jun 28;8:45. doi: 10.1186/1743-7075-8-45 (PMC3152873; doi:10.1186/1743-7075-8-45)
Supplement: Additional file 3 — Continuous treatment effect of L-tryptophan on hepatic gene expression levels expressed as relative changes determined by quantitative RT-PCR. Effect of continuous treatment of L-Trp on hepatic mRNA expression levels of Gck, Pck1, Fbp1, and Pklr [file 1743-7075-8-45-S3.DOC]

Table S3. Continuous treatment effect of l-tryptophan on hepatic gene expression levels expressed as relative changes determined by quantitative RT-PCR.^1^

| Gene | Control (n=4)  (ME ± SEM) | LT200 (n=4)  (ME ± SEM) | LT1000 (n=4)  (ME ± SEM) |
| --- | --- | --- | --- |
| *Gck* | 1.0 ± 0.4 | 1.9 ± 0.6 | 0.9 ± 0.1 |
| *Pck1* | 1.0 ± 0.2 | 1.1 ± 0.1 | 1.4 ± 0.2 |
| *Fbp1* | 1.0 ± 0.1 | 1.5 ± 0.3 | 1.0 ± 0.1 |
| *Pklr* | 1.0 ± 0.2 | 1.3 ± 0.2 | 1.4 ± 0.2 |

^1^ mRNA expression (fold). *Gck*, glucokinase; Pck1, phosphoenolpyruvate carboxykinase 1; *Fbp1*, fructose bisphosphatase 1; *Pklr*, liver-type pyruvate kinase. LT200, diet supplemented with 200 mg⋅kg^-1^ l-tryptophan; LT1000, diet supplemented with 1000 mg⋅kg^-1^ l-tryptophan.
